# Adipose Stem Cell-Seeded Decellularized Porcine Pericardium: A Promising Functional Biomaterial to Synergistically Restore the Cardiac Functions Post-Myocardial Infarction

**DOI:** 10.3390/vetsci10110660

**Published:** 2023-11-17

**Authors:** Hussein M. El-Husseiny, Eman A. Mady, Tatsuya Usui, Yusuke Ishihara, Toshinori Yoshida, Mio Kobayashi, Kenta Sasaki, Danfu Ma, Akira Yairo, Ahmed S. Mandour, Hanan Hendawy, Ahmed S. Doghish, Osama A. Mohammed, Ken Takahashi, Ryou Tanaka

**Affiliations:** 1 Laboratory of Veterinary Surgery, Department of Veterinary Medicine, Faculty of Agriculture, Tokyo University of Agriculture and Technology, 3-5-8 Saiwai Cho, Fuchu-shi 183-8509, Tokyo, Japan; vm.knt.sasaki@gmail.com (K.S.); dandanma1000@gmail.com (D.M.); akirayairo@gmail.com (A.Y.); dr_mandour@vet.suez.edu.eg (A.S.M.); hanan_attia@vet.suez.edu.eg (H.H.); 2 Department of Surgery, Anesthesiology, and Radiology, Faculty of Veterinary Medicine, Benha University, Moshtohor, Toukh 13736, Elqaliobiya, Egypt; 3 Laboratory of Veterinary Physiology, Department of Veterinary Medicine, Faculty of Agriculture, Tokyo University of Agriculture and Technology, 3-5-8 Saiwai Cho, Fuchu-shi 183-8509, Tokyo, Japan; s211566z@st.go.tuat.ac.jp; 4 Department of Animal Hygiene, Behavior and Management, Faculty of Veterinary Medicine, Benha University, Moshtohor, Toukh 13736, Elqaliobiya, Egypt; 5 Laboratory of Veterinary Pharmacology, Department of Veterinary Medicine, Faculty of Agriculture, Tokyo University of Agriculture and Technology, 3-5-8 Saiwai Cho, Fuchu-shi 183-8509, Tokyo, Japan; fu7085@go.tuat.ac.jp (T.U.); ishiharatat@gmail.com (Y.I.); 6 Laboratory of Veterinary Pathology, Division of Animal Life Science, Institute of Agriculture, Tokyo University of Agriculture and Technology, 3-5-8 Saiwai-cho, Fuchu-shi 183-8509, Tokyo, Japan; yoshida7@cc.tuat.ac.jp (T.Y.); s212838s@st.go.tuat.ac.jp (M.K.); 7 College of Veterinary Medicine, Nanjing Agricultural University, No. 1 Wei-Gang, Xuanwu District, Nanjing 210095, China; 8 Department of Animal Medicine (Internal Medicine), Faculty of Veterinary Medicine, Suez Canal University, Ismailia 41522, Ismailia, Egypt; 9 Department of Veterinary Surgery, Faculty of Veterinary Medicine, Suez Canal University, Ismailia 41522, Ismailia, Egypt; 10 Department of Biochemistry, Faculty of Pharmacy, Badr University in Cairo (BUC), Badr City 11829, Cairo, Egypt; ahmed_doghish@azhar.edu.eg; 11 Department of Biochemistry, and Molecular Biology Faculty of Pharmacy (Boys), Al-Azhar University, Nasr City 11651, Cairo, Egypt; 12 Department of Clinical Pharmacology, College of Medicine, University of Bisha, Bisha 61922, Saudi Arabia; oamohamed@ub.edu.sa; 13 Department of Pediatrics and Adolescent Medicine, Juntendo University Graduate School of Medicine, Bunkyo 113-8421, Tokyo, Japan; kentaka@juntendo.ac.jp

**Keywords:** adipose stem cells, decellularized porcine pericardium, DPP, biomaterials, tissue engineering, cell delivery, stem cells, heart, intraventricular pressure gradient, IVPG, echocardiography

## Abstract

**Simple Summary:**

Over the years, diverse therapeutic protocols have been employed for cardiac tissue engineering (CTE) and improvement of the heart functions. Among them are stem cells which possess a high tissue-regeneration potential. However, the optimum delivery protocol with effective cell retention and homing at the target site is still challenging. Hence, a wide range of natural and synthetic biomaterials have been utilized as cell delivery cargoes to address these limitations. Natural decellularized biomaterials could provide successful biocompatible, biodegradable, elastic, and strong platforms that avoid the drawbacks of synthetic ones. Hence, in the present study, we have prepared a decellularized porcine pericardium (DPP) patch to provide successful tailored cell discharge, retention, and homing at the site of the myocardial infarction (MI) with an outstanding ability to restore the damaged heart activity. Moreover, we have provided for the first time a novel echocardiographic-derived imaging modality called the intraventricular pressure gradient (IVPG) to evaluate the process. The IVPG provides a precise, non-invasive, and facile tool to assess cardiac functions. This speeds up the accumulation of knowledge and opens new avenues to objectively evaluate diagnosis and treatment, which encourages the implementation of such a cutting-edge strategy in clinical practices in the near future.

**Abstract:**

Myocardial infarction (MI) is a serious cardiovascular disease as the leading cause of death globally. Hence, reconstruction of the cardiac tissue comes at the forefront of strategies adopted to restore heart functions following MI. In this investigation, we studied the capacity of rat adipose-derived mesenchymal stem cells (r-AdMSCs) and decellularized porcine pericardium (DPP) to restore heart functions in MI animals. MI was induced in four different groups, three of which were treated either using DPP (MI-DPP group), stem cells (MI-SC group), or both (MI-SC/DPP group). Cardiac functions of these groups and the Sham group were evaluated using echocardiography, the intraventricular pressure gradient (IVPG) on weeks 2 and 4, and intraventricular hemodynamics on week 4. On day 31, the animals were euthanized for histological analysis. Echocardiographic, IVPG and hemodynamic findings indicated that the three treatment strategies shared effectively in the regeneration process. However, the MI-SC/DPP group had a unique synergistic ability to restore heart functions superior to the other treatment protocols. Histology showed that the MI-SC/DPP group presented the lowest (*p* < 0.05) degeneration score and fibrosis % compared to the other groups. Conclusively, stem cell-seeded DPP is a promising platform for the delivery of stem cells and restoration of heart functions post-MI.

## 1. Introduction

Cardiovascular diseases are the number one cause of death worldwide [1]. Among them is myocardial infarction (MI) which is the leading cardiac affection that causes significant cardiac dysfunctions with possible heart failure and death. Despite the significant advances in medical and surgical treatments, undesirable life-threatening sequels of MI can develop including ischemic cardiomyopathy [2]. Recently, cardiac tissue regeneration has been reported as a potential option to restore heart functions post-MI [3,4]. Moreover, the use of adipose-derived mesenchymal stem cells (AdMSCs) in this track is highly promising. They were found to enhance the neovascularization of the damaged heart tissue via several growth factors such as basic fibroblast growth factor (bFGF), vascular endothelial growth factor (VEGF), and hepatocyte growth factor (HGF) [5,6]. Furthermore, they share effectively to restore heart functions with ameliorated heart remodeling and contractility [7,8]. Over the years, many investigations have been conducted to amend the usefulness of AdMSC-based therapies incorporating cell genetic engineering to improve cell survival and homing [9,10]. Lately, the field of tissue engineering has also raised great promise in utilizing diverse technologies and materials to revitalize injured tissues [11]. The emergence of novel natural or synthetic biomaterials has received great attention for cardiac tissue engineering (CTE) [12,13,14,15,16,17,18]. Despite their ability to afford topical mechanical support to the damaged tissues and to modify the pathway of remodeling, the utilization of the synthetic scaffolds for CTE is still not the optimum choice, attributed to their limited potential to share effectively in the regeneration process, restricted capacity to grow with the tissue, and lack of active contractility [19,20]. Hence, the natural decellularized extracellular matrix (ECM) derived from diverse body tissues such as the pericardium [21,22,23], urinary bladder [24], stomach [25], small intestines [26], amniotic membrane [27], and others was introduced as a markedly auspicious choice for CTE [28]. The constituents of the ECM were shown to offer a favorable microenvironment for the delivery of the loaded stem cells to the targeted site of MI [29,30]. Several animal studies have been accomplished using autologous materials to restore cardiac functions. However, their limited availability renders it a significant challenge to utilize them on a large scale. Thus, the strategy to decellularize the xenografts was found to be encouraging as it provokes a lower immune reaction and improves the recellularization ability as they enhance the adhesion, immigration, and growth of the cells [31,32,33]. To improve the outcomes from the use of biomaterials, recellularization and the remodeling after seeding the decellularized xenografts with MSCs has been evaluated [34]. One of the important characteristics of the MSCs is their ability to exhibit relatively little MHC class I and II expression [35]. The incorporation of the cells inside the natural scaffold is superior to direct cell injection to treat the MI in terms of minimizing the unfavorable milieu of the MI, and amelioration of cell survival and retention [36,37]. To evaluate the ability of these cell-seeded materials to restore the heart functionalities, diverse approaches have been employed. Echocardiography is the gold standard technique in this regard being a non-invasive and safe technique where the hazards due to exposure to radiation are avoided especially when serial checking is necessary [38]. Recently, the intraventricular pressure gradient has presented a novel echocardiographic-derived imaging modality to precisely assess heart functions [39,40,41]. In addition to the merits of echocardiography, the IVPG could provide a precise assessment of the heart function earlier than conventional echocardiography as it can detect the alterations in the intraventricular blood flow and pressure before the structural alterations become evident [40]. We believe that this is the first report to use the IVPG to assess heart functions after cell- and biomaterial-based therapies. Hence, the main objectives of the present study are to assess the behavior of the decellularized porcine pericardium (DPP) as an acellular ECM seeded with r-AdMSCs for the regeneration of infarcted heart tissue and to investigate the utility of the IVPG to evaluate the restored heart functions.

## 2. Materials & Methods

### 2.1. Animals of the Study

Thirty male Sprague Dawley (SD) rats (Kitayama Labes Co., Ltd., Nagano, Japan), 3 months of age, 376.30 ± 27.24 g body weight were used. Animals were housed separately at 25 ± 2 °C with a 12-h light/dark cycle with unlimited access to food and water. The Institutional Animal Care and Use Committee of Tokyo University of Agriculture and Technology evaluated and approved all procedures (Approval No. R05-91), which were carried out following the Guide for the Care and Use of Laboratory Animals issued by the US National Institutes of Health (NIH).

### 2.2. Study Design

**Figure 1:**
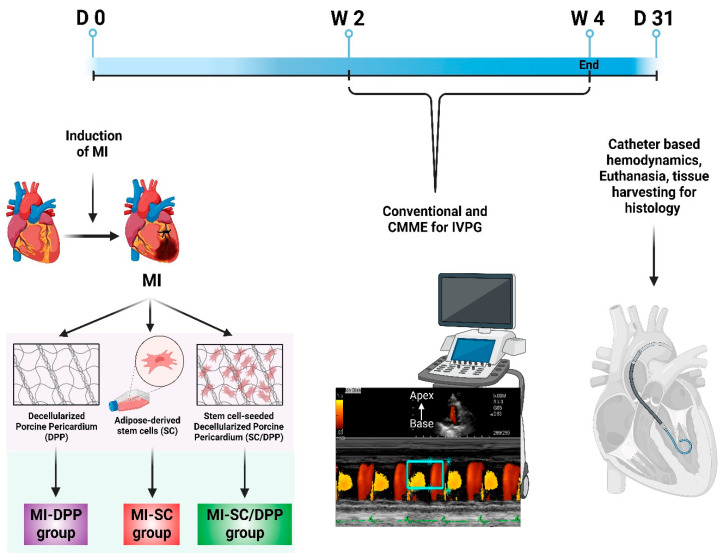
Schematic illustration of the current study design.

The animals were assigned to one of five groups (n = 6/group). MI was induced in four groups, three of which received diverse treatment protocols. The first group included animals with thoracotomy without induction of MI (Sham group). In the second group, MI was induced surgically without receiving any treatment (MI group). In the third group, MI animals were implanted with decellularized porcine pericardium (MI-DPP). The fourth group included animals with MI injected with r-AdMSCs (MI-SC). The last group comprised animals with MI treated with stem cell-seeded DPP (MI-SC/DPP). Conventional transthoracic and color M-Mode echocardiography (CMME) for IVPG were assessed in all groups on weeks 2 and 4 post-surgery. On day 91, hemodynamics (indices of intraventricular pressure (IVP)) were recorded under the effect of inhalation anesthesia using 1.5–2% isoflurane (Figure 1). Afterwards, animals were euthanized with isoflurane overdose, and the harvested hearts were histologically examined.

### 2.3. Isolation and Characterization of the r-AdMSCs

Inguinal adipose tissue was harvested from five healthy, 250–300 gm in weight, and two months in age SD rats under strict sterile conditions. Then, r-AdMSCs were isolated according to our recently published protocol [5]. Briefly, the collected inguinal fat depots were cleaned well with PBS, thoroughly minced, and digested at 37 °C for 1 h using 0.1% (*w*/*v*) collagenase type 1 (1 mg/mL; Gibco by Life Technologies, Waltham, MA, USA). After centrifugation (800× *g* for 10 min), filtration by 100 µm filters, and RBCs lysis, the obtained cell pellets were suspended in Dulbecco’s Modified Eagle Medium (DMEM, 043-30085, Fujifilm Wako Pure Chemical Corporation, Osaka, Japan). Isolated cells were cultured on 75 cm^2^ culture dishes (5 × 10^5^ cells/dish), nourished by DMEM containing fetal bovine serum (FBS) 10%, and incubated at 37 °C, 5% CO_2_, and 95% humidity. The medium was changed every 3 days until it reached 80% confluence where cells were passaged. We used the third passage cells (P3) for the in vivo cardiac regeneration application. The morphological characterization, immunophenotypic characterization (flowcytometry), pluripotency, and multi-lineage (adipogenic, osteogenic, and chondrogenic) differentiation potential of the isolated inguinal r-AdMSCs were assessed and elaborated in detail in our recent investigation [5].

### 2.4. Decellularization and Characterization of DPP Biomaterial

Decellularized Porcine Pericardium (DPP) biomaterials were prepared according to our recently published work [6] using a protocol modified after Ramm. R. et al., 2020 [42]. In brief, native PP tissues were obtained from healthy 5–6 months old pigs of both sexes and purchased from one of Tokyo’s local slaughterhouses. Then, in the laboratory, the attached fat and surplus connective tissue were eliminated. The tissues were cleaned with PBS and disinfected with sodium Braunol^®^ (B. Braun) for 5 min under shaking. Decellularization was accomplished via incubation of the tissues in Trypsin 0.125% − EDTA 0.05% (Trypsin/EDTA, T4049-100 ML, Sigma Aldrich, St. Louis, MO, USA) for 90 min, then Triton X (TX) 0.5% (TX-100, T8787-100 ML, Sigma Aldrich, St. Louis, MO, USA) (2 cycles/12 h each). Afterward, tissues were incubated in sodium dodecyl sulfate 0.5% (SDS, L3771, Sigma Aldrich, St. Louis, MO, USA) (2 cycles/12 h each), washed by water twice (12 h/cycle), rinsed by PBS (14 cycles/12 h each), and preserved in PBS comprising penicillin 1 mg/mL, amphotericin 1 mg/mL, and streptomycin 1 mg/mL antibiotics at 4 °C till their use. All steps of incubation were performed under shaking with an orbital shaker (200 rpm) at room temperature. Assessment of DPP biomaterials structure, biomechanics, and in vitro and in vivo biocompatibility was accomplished and comprehensively described in our previous study [6].

### 2.5. Preparation of Stem Cell-Seeded DPP

Before seeding, DPP sheets were taken from the sterile containers and cut into 1.5 × 1.5 cm^2^ pieces. The DPP patches were cultured with DMEM culture media for 24 h in culture plates. Then, the prepared inguinal r-AdMSCs were seeded on the DPP according to a method described before [21,43]. Briefly, the r-AdMSCs (1.0 × 10^6^ cells) were suspended in 50 μL of the culture medium and seeded over the DPP patches for 30 h. Then, the drops of the culture medium were added and further cultured for 90 min. After that, we added 20 mL of the culture medium to the culture plates. The DPP patches seeded with the stem cells were cultured at 37 °C in 5% CO_2_ and 95% humidity for 5–7 days before implantation.

### 2.6. Induction and Treatment of Myocardial Infarction

Procedures to induce MI were previously detailed [44]. Following anesthesia, intubation, and ventilation with oxygen and 1.5–2% isoflurane mixture using a rodent-designed ventilator (Harvard Apparatus, Boston, MA, USA), the left anterior descending (LAD) artery was exposed through a left lateral thoracotomy incision at the level of the 4th intercostal space. LAD was ligated using prolene 6-0 suture 4–5 mm below the atrioventricular junction. MI was verified by the pallor of the left ventricle (LV) below the ligation site. Following the induction of MI, animals in the last three groups were treated [45,46]. MI-DPP group was transplanted using DPP on the infarct area. In the MI-SC group, 100 μL of r-ADMSCs (1.0 × 10^6^ per rat) was injected carefully in the peripheries and the center of the infarct site. MI-SC/DPP group was treated via transplantation of the infarct site by the stem cell-seeded DPP.

### 2.7. Cardiac Function Assessment

To assess the capacity of stem cell-seeded DPP to restore heart functions after MI, we performed conventional echocardiography, CMME-derived IVPG, and catheter-based hemodynamic (Figure 1).

#### 2.7.1. Conventional Echocardiography

Conventional echocardiography was accomplished after the guidelines of the American Society of Echocardiography (ASE) [47] and Zacchigna S. et al. [48]. ProSound 7 ultrasound system (Hitachi-Aloka Medical Ltd., Tokyo, Japan) operated with a 12 MHz transducer reinforced with CMME and concurrent ECG was used. More details about echocardiographic assessments were described in detail in our previous works [40].

#### 2.7.2. Intraventricular Pressure Gradient (IVPG)

The CMME was utilized to calculate IVPG according to the technique comprehensively explained in our earlier investigations. The sweep speed was set to 300 mm/s, and the baseline shift of the color was adjusted to −64 to elevate the limit of Nyquist for proper tracing of the CMME. Then, the CMME images were analyzed by MATLAB (The MathWorks, Natick, MA, USA) to attain the spatial and temporal IVPG profiles. IVPG was measured at 4 portions along the length of the LV: basal, mid-, mid-to-apical (MA), and apical IVPG. Total IVPG represents the summation of these segmental pressures.

#### 2.7.3. Intra-Ventricular Catheter-Based Hemodynamics Analysis

On day 31, under the effect of inhalation anesthesia, a 2-Fr microtip pressure catheter (SPR-407, Mikro-Cath, Millar Instruments, Houston, TX, USA) was retrogradely inserted via the right carotid artery till reaching the LV to record the intraventricular pressure and its derived hemodynamic indices. To prevent fluctuations caused by breathing, the respirator was momentarily turned off while the data were being recorded. Acquisition of the measures was accomplished over 10–15 heart cycles and data were then averaged. Acquired data were analyzed using LabChart Pro (LabChart v8, AD Instruments, Colorado Springs, CO, USA) software [40].

### 2.8. Histological Evaluations

Following euthanasia, the extracted heart tissues from diverse groups of the present study were washed using PBS and fixed in 4% buffered formalin. Then, the tissues were embedded in paraffin and cut into 5 μm thick sections. Afterward, deparaffinization was accomplished in xylene followed by dehydration in gradually elevated concentrations of ethanol mixtures and staining using hematoxylin and eosin (H&E) to check the structure of the heart. To evaluate the regeneration capacity of the treatment options, ten H&E-stained sections from each group were blindly evaluated by a histopathologist for inflammatory (mononuclear) cell infiltration, the existence of fibrosis (necrotic tissue), interstitial edema, in addition to evaluation of the myocytes’ assembly and arrangement of the cardiomyocytes. The findings in each group were scored according to [49] where no changes (0), mild changes (1), moderate changes (2), severe changes (3), or extremely severe changes (4) scores have been adopted. To assess the fibrosis area % in different groups, Masson’s trichrome (MTC) staining was performed. NIH Image J software was utilized to measure the fibrosis % (Fibrosis % = (fibrosis area/LV wall area) × 100) [46].

### 2.9. Statistical Analysis

Data were displayed as mean ± SD. Values of the conventional echocardiography and CMME-derived IVPG between various groups at W2 and W4 were compared using a two-way analysis of variance (ANOVA) and Tukey’s post hoc test. On the other side, the hemodynamic indices, histological scores, and fibrosis area % measured at the end of the present study in diverse groups were compared by Kruskal–Wallis one-way analysis of variance (ANOVA) and Dunn’s post hoc test. GraphPad Prism software version 9 (GraphPad Software, Inc., La Jolla, CA, USA) was exploited to perform the statistics, and *p* < 0.05 was identified as statistically significant.

## 3. Results

In the present study, both DPP and adipose stem cells presented an ability to restore and improve heart functions after MI. However, the merge of both DPP and adipose stem cells in one therapeutic protocol showed a synergistic capacity to restore heart functions in an augmented manner. This was confirmed using echocardiographic, IVPG, hemodynamic, and histological evaluations.

### 3.1. Echocardiography

**Figure 2:**
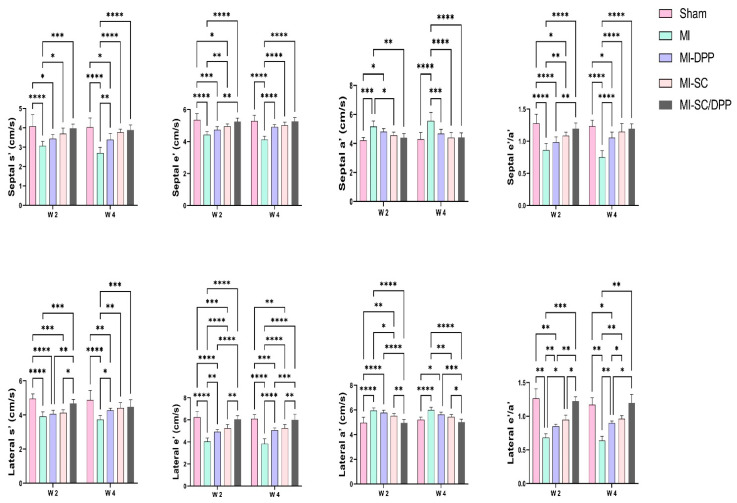
Tissue Doppler Imaging (TDI) echocardiography to assess the role of the stem cell-seeded biomaterial in the restoration of the diastolic functions following myocardial infarction (MI). Data are presented as means ± SD. * *p* < 0.05, ** *p* < 0.01, *** *p* < 0.001, and **** *p* < 0.0001.

**Figure 3:**
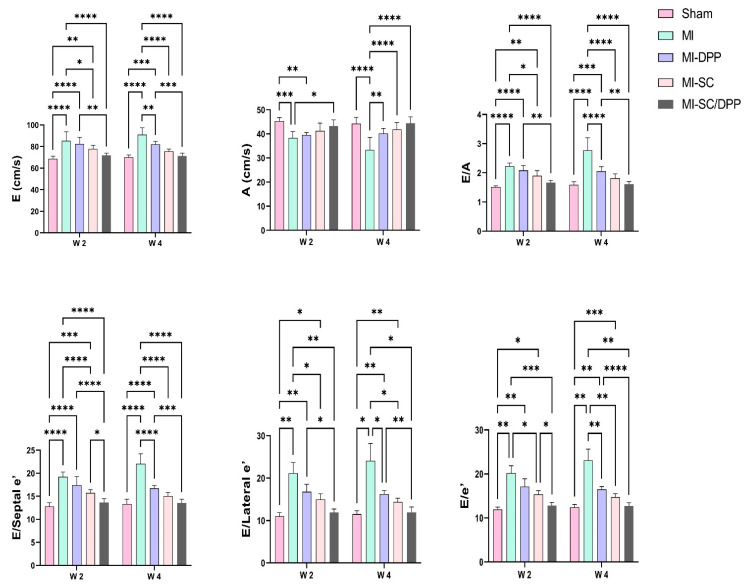
Transmitral flow echocardiography to evaluate the potency of the stem cell-seeded biomaterial in the restoration of the diastolic functions following myocardial infarction (MI). Data are presented as means ± SD. * *p* < 0.05, ** *p* < 0.01, *** *p* < 0.001, and **** *p* < 0.0001.

The alterations encountered in the echocardiographic values are presented in Table 1 and Figure 2 and Figure 3. Echocardiographic evaluation of heart function revealed that both the biomaterial (MI-DPP group) and the stem cells (MI-SC) played an important role in partially restoring cardiac function and preventing the severe devastating effect of MI on the cardiac muscle. However, the stem cell-seeded biomaterial (MI-SC/DPP group) exhibited the highest ability to regenerate the damaged cardiac muscle and preserve its functionality. Moreover, their influence improved over time. Generally, there was no substantial change amongst the MI-DPP and MI-SC groups except for the marked rise of the LV internal diameter during systole (LVIDs) and the decrement of the fractional shortening (FS) on W2 in the MI-DPP group compared to the MI-SC group (Table 1). There was a marked reduction in the LV internal diameter during diastole (LVIDd), LVIDs, the LV posterior wall diameter during diastole (LVPWd), the LV posterior wall diameter during systole (LVPWs), and the LV mass (LVM) in the MI-SC/DPP group on W4 compared to the MI group (*p* < 0.0001) and the MI-DPP group (*p* < 0.01). Similar findings were observed on W2 without significant variations in the LVPWd and the LVM. On the other side, the intraventricular septal thickness during diastole (IVSd), intraventricular septal thickness during systole (IVSs), ejection fraction (EF), and FS were markedly elevated in the MI-SC/DPP group in comparison to the MI and the MI-DPP groups on W4. The same was noticed on W2 without significant change in the IVSs between these groups. As illustrated in Figure 2 and Figure 3, the analysis of the diastolic functions showed that the tissue Doppler Imaging (TDI) was altered in diverse groups. The LV wall velocity at systole (s′), LV wall velocity at early diastole (e′), and LV early to late diastolic velocity (e′/a′) on the septal and lateral sides markedly increased in the treated animals compared to the MI group. The septal LV wall velocity at late diastole (a′), and lateral a′ markedly decreased in the MI-DPP, MI-SC, and MI-SC/DPP groups in comparison to the MI animals over the various periods of the investigation (Figure 2). Regarding the transmitral flow, the early diastolic transmitral flow velocity (E) and early to late diastolic transmitral flow velocities ratio (E/A) decreased significantly in the MI-SC/DPP group in comparison to the MI and the MI-DPP groups on W2 and W4. However, the late diastolic transmitral flow velocity (A) wave was increased in the MI-SC/DPP group. No marked change was encountered in the trans-mitral flow parameters among the MI-SC/DPP and the MI-SC groups throughout the study. The E/septal e′, E/lateral e′, and E/e′ notably declined in the treatment groups versus the MI animals throughout the study (Figure 3).

### 3.2. The Intraventricular Pressure Gradient (IVPG)

**Figure 4:**
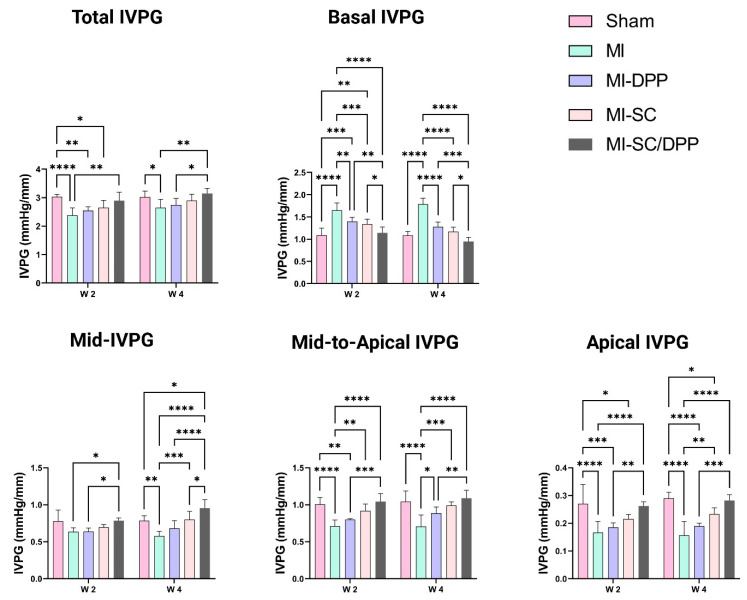
Intraventricular pressure gradient to evaluate the ability of the capacity of the stem cell-seeded biomaterial in the restoration of the diastolic functions following myocardial infarction (MI). Data are means ± SD. * *p* < 0.05, ** *p* < 0.01, *** *p* < 0.001, and **** *p* < 0.0001.

The function of the stem cell-seeded biomaterial in the restoration of the heart integrity assessed by the CMME-derived IVPG is exhibited in Figure 4. The results showed that changes were found in both the total and segmented IVPG measures. The treated groups had higher total, mid-, MA, and apical IVPGs than those of the MI group. In contrast, the basal IVPG decreased in the treated animals compared to the MI animals. Regarding total IVPG, MI-SC/DPP animals showed a significantly higher total IVPG compared to the MI group on W2 and to the MI and MI-DPP groups on W4. There was no substantial alteration in the total IVPG between the MI-SC and the MI-SC/DPP groups throughout the current work. Regarding the segmental IVPGs, there was a pronounced diminution in the basal IVPG in the MI-SC/DPP animals compared to the MI, MI-DPP, and MI-SC groups in the study. Moreover, the basal IVPG was considerably lesser in the MI-DPP and MI-SC groups versus the MI group on W2 and W4. The mid-IVPG was obviously (*p* < 0.05) greater in the MI-SC/DPP group than that of the MI and MI-DPP groups on W2 and W4 (*p* < 0.05, *p* < 0.0001, respectively). No considerable change was observed in the mid-IVPG on W2 between the MI-SC/DPP and MI-SC groups on W2. However, it was markedly higher (*p* < 0.05) on W4 between the MI-SC/DPP and the MI-SC groups. Likewise, the MA IVPG was drastically more elevated in the MI-SC/DPP animals than in the MI and MI-DPP animals. In addition, it was elevated markedly in the MI-SC group compared to the MI group all over the study. No change was detected among the MI-SC/DPP and MI-SC groups. The apical IVPG increased extensively in the MI-SC/DPP group in comparison to the MI and MI-DPP groups on W2 and W4. No significant change was encountered in the apical IVPG between the MI-SC and the MI-SC/DPP groups and between the MI-SC and the MI-SC/DPP groups at different points of the experiment. Also, a significant rise (*p* < 0.01) of the apical IVPG was detected in the MI-SC group versus the MI group (Figure 4).

### 3.3. Intra-Ventricular Catheter-Based Hemodynamics Analysis

**Figure 5:**
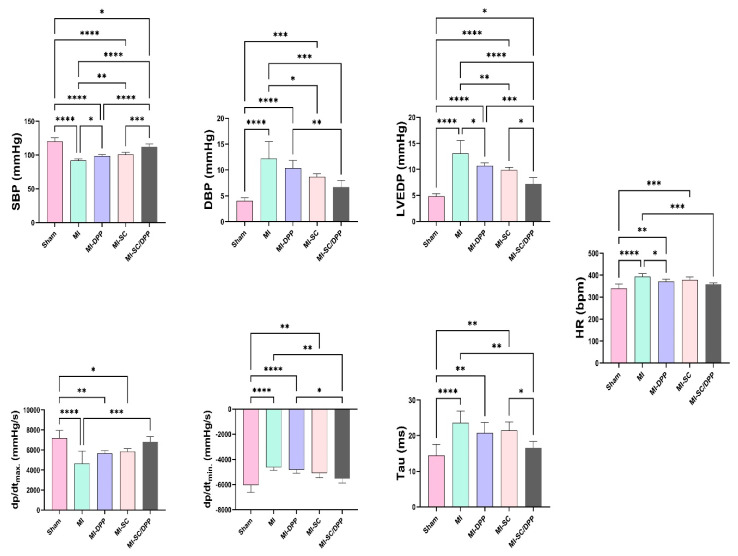
Intraventricular catheter-based hemodynamic indices in different groups with and without treatment of myocardial infarction. Data are presented as means ± SD. * *p* < 0.05, ** *p* < 0.01, *** *p* < 0.001, and **** *p* < 0.0001.

The variations in the intraventricular pressure-derived measures between the diverse groups at the end of the present study are displayed in Figure 5. The maximal (systolic) blood pressure (SBP) was considerably greater in the MI-SC/DPP animals than in the MI, MI-DPP, and MI-SC groups. It was substantially lesser in the MI group versus the sham, MI-DPP, and MI-SC groups. No significant alteration was found in the SBP between the MI-DPP and MI-SC groups. The positive LV pressure derivative (dP/dt_max._), and negative LV pressure derivative (−dP/dt_min._) presented a marked increase in the MI-SC/DPP group compared to the MI group. Moreover, the −dP/dt_min._ was significantly higher in the MI-SC/DPP group than in the MI-DPP group (*p* < 0.05). No marked change was observed in the ±dp/dt between the MI-SC and MI-SC/DPP groups. The LV end-diastolic pressure (LVEDP) was notably greater in the MI animals compared to the sham, MI-DPP, MI-SC, and MI-SC/DPP groups (*p* < 0.0001, *p* < 0.05, *p* < 0.01, *p* < 0.0001, respectively). Moreover, it was markedly lower in the MI-SC/DPP animals versus the MI-DPP and the MI-SC animals. The time constant of isovolumic relaxation (Tau (τ)) value was also lower in the MI-SC/DPP animals in comparison to the MI-SC and the MI animals. Comparison between the MI-DPP and MI-SC groups revealed a considerable alteration among the MI-SC and the MI-DPP groups in the LVEDP measure and Tau index. There was a marked rise in the heart rate (HR) in the MI animals versus the others. Despite there being no noteworthy modification in the HR between the MI-DPP, MI-SC, and MI-SC/DPP groups, it was drastically greater in the MI-DPP and MI-SC animals versus the sham group (Figure 5).

### 3.4. Histological Evaluations

**Figure 6:**
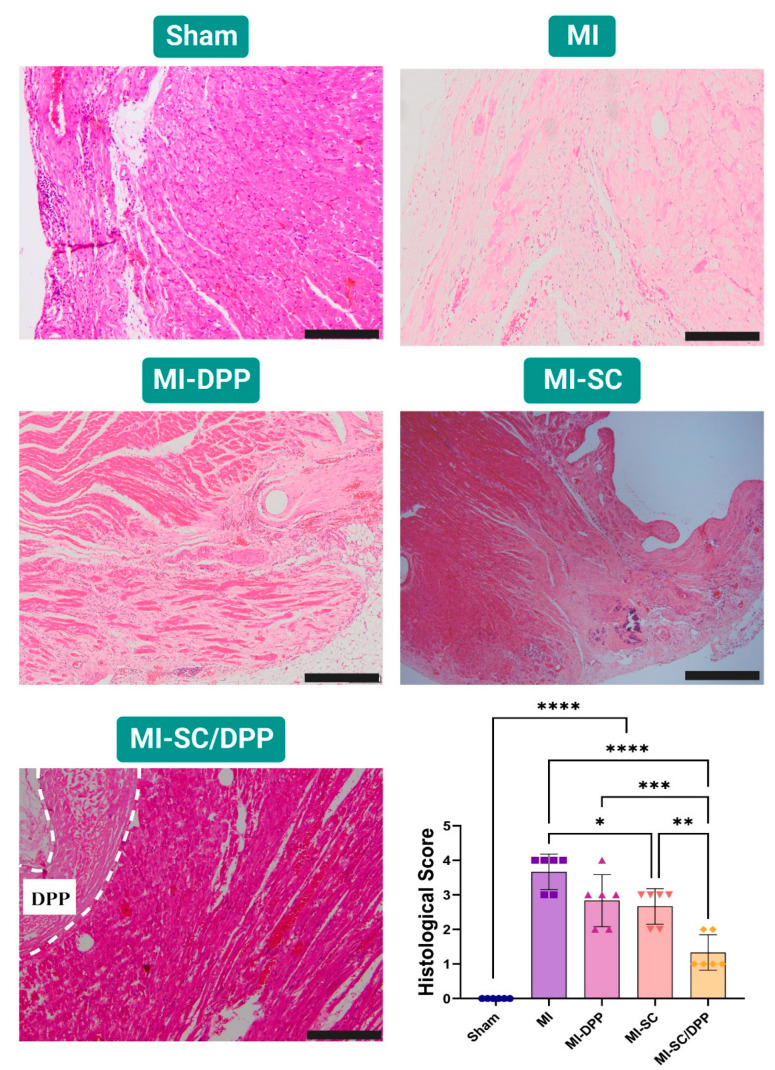
Histological assessment of the heart tissues using Hematoxylin and Eosin (H&E) stain in diverse groups of the present study 4 weeks post-surgery. The bar graph presented the quantitative histological score in different groups. Scale bar: 200 µm. Data displayed as mean ± SD. * *p* < 0.05, ** *p* < 0.01, *** *p* < 0.001, and **** *p* < 0.0001.

**Figure 7:**
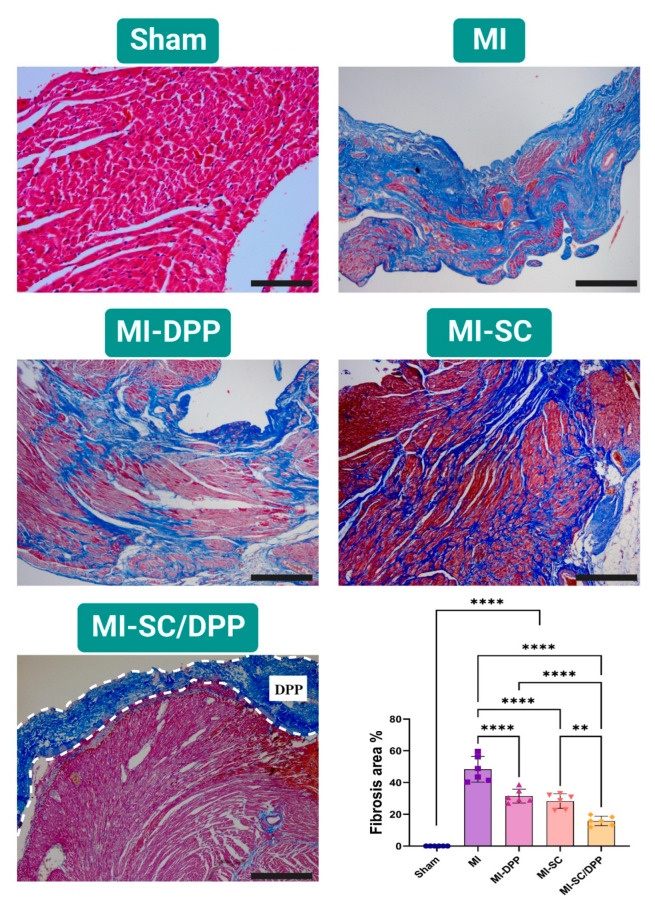
Representative images of Masson’s trichrome (MTC) stained hearts 4 weeks post-surgery in various groups of the present study. The bar graph exhibited the quantitative assessment of the fibrosis area % in various groups. Scale bar: 200 µm. Data displayed as mean ± SD. ** *p* < 0.01, and **** *p* < 0.0001.

The histopathological evaluations of the hearts isolated from different groups on W4 are illustrated in Figure 6 and Figure 7. H&E staining confirmed the normal unaltered cardiac tissue structure in the sham-operated group. On the contrary, the MI animals presented elevated inflammatory cell infiltration, extensive fibrosis, and obvious interstitial edema among the cardiomyocytes. Moreover, the cardiomyocytes exhibited disassembly with distorted fibers. These alterations (inflammation, fibrosis, and interstitial edema) were markedly lower in the treatment groups in variable degrees. Histological scores confirmed that the treatment groups shared in the regeneration process and lowered the remodeling process effectively as they had significantly lower scores compared to the MI group (3.67 ± 0.47, 2.83 ± 0.69, 2.67 ± 0.47, 1.33 ± 0.47 in the MI, MI-DPP, MI-SC, and MI-SC/DPP groups, respectively). The MI-SC and MI-SC/DPP groups had lower scores than the MI-DPP group, and the lowest scores were observed in the MI-SC/DPP group (Figure 6). MTC staining showed that the intensity of fibrosis indicated by blue-colored collagen fibers was very high in the MI group and lower in the treatment groups with the lowest fibrosis recorded in MI-SC/DPP group. The fibrosis area % considerably declined in the MI-SC/DPP animals versus the others (15.83 ± 2.72, 28.37 ± 4.22, 31.5 ± 4.01, 48.33 ± 7.38, and 0.00 ± 0.00 in MI-SC/DPP, MI-SC, MI-DPP, MI, and Sham groups, respectively) (Figure 7).

## 4. Discussion

In the present study, we aimed to provide a potent r-ADMSCs seeded DPP scaffold as a promising therapeutic modality for CTE. Cell transplantation is a promising therapeutic choice that patients with ischemia and ventricular dysfunction may benefit from to restore cardiac function. However, cases with a large and developed scar do not benefit from cell transplantation alone, which is attributed to the constrained engraftment process [21]. Compared to the MI group, both the MI-DPP and MI-SC groups could share in the regeneration of the degenerated cardiac muscles. That was evidenced by the partial improvement of the conventional echocardiographic structural (LVIDd, LVIDs, IVSd, IVSs, LVPWd, LVPWs, and LVM) and functional findings (EF, and FS). On the other hand, the diastolic functions (E, A, E/A, E/e′, as well as lateral and septal TDI measures) also presented a marked progress in these groups in comparison to the MI animals. These findings are in line with those reported before confirming the regeneration power and cardiac function restoration ability of these therapeutic modalities [45,50,51,52]. The stem cells play a key role in the enhancement of neovascularization via prolonged production of valuable paracrine factors, but their limited retention and engraftment are still encountered as a significant challenge [45]. Various approaches have been adopted for cell delivery to the infarct site including intravenous infusion [53], intracoronary injection [54], and direct epicardial [55] or endocardial injection via a catheter [52,56]. These cell delivery strategies have presented a limited capacity for cell retention as well as homing [50]. Hence, the decellularized biomaterial used in the present study holds promise for the healing of cardiac tissue. Proteins, glycosaminoglycans, and proteoglycans are among the components found in normal tissues that the dECM most successfully preserves, making it the perfect support for the regeneration and repair of injured myocardium as they are optimal for cell adhesion, growth, and multiplication of cardiac cells [16,57]. Using dECM-based patches either alone or in combination with cells and/or other GFs, stem cells, or polymers can stimulate regeneration and restore the cardiac function [58]. This is supported by the immigration of numerous host cells to the dECM and the development of several blood vessels 7 days after transplantation [59]. Compared to cell aggregates, cells that were embedded in dECM, such as hiPSC-CMs, showed more electrical activity and maturation. The cardiac patch reduced the infarct area and increased EF after being implanted in the infarct location [60]. In the present study, stem cell-seeded DPP presented a superior capacity over the stem cells alone or the patch alone to achieve potent CTE following MI. This is consistent with many previous studies [21,43,45]. This synergistic effect was obvious through the findings described in our investigation. Two-dimensional scaffolds and patches have been reported to provide outstanding cell delivery and retention at the site of MI. Moreover, they could provide elastic support with or without cells along the exterior ventricle wall of the myocardium for regeneration [50]. After being applied to a rat with acute MI, our cardiac patches enhanced the left ventricular function. The patch was still functional 4 weeks after implantation. A previous investigation revealed that cells in the engineered human cardiac patches survived much better and longer after transplantation than those obtained by direct myocardial injection of suspended cells that are well known to typically die and disappear several weeks after direct injection [61]. Hence, this study’s successful treatment of MI using r-AdMSCs seeded DPP cardiac patches may be closely related to the extended cell survival after transplantation. The results of the IVPG indices, the hemodynamic indices, and the histological assessments confirm this assumption. Researchers established a correlation between cardiac motility and the MA IVPG. Meanwhile, the E wave velocity correlated with the basal IVPG [62]. According to the most recent guidelines for LV diastolic function, E/e′ is an essential component of the staging strategy for diastolic dysfunction [63]. To our knowledge, the present investigation proved, for the first time, the feasibility of the IVPG as a novel non-invasive CMME-derived imaging modality to assess heart function restoration following MI treatment using stem cell-seeded decellularized biomaterial.

## 5. Conclusions

Conclusively, the stem cell-seeded DPP biomaterials presented a synergistic capacity to efficiently regenerate the heart tissue and restore cardiac functions post-MI superior to the single use of the stem cells or the DPP biomaterial. Moreover, the IVPG was found to be a promising precise novel imaging modality to assess the cardiac functions of post-infarction regenerated hearts.

## Figures and Tables

**Table 1:** Cardiac function assessment using conventional echocardiography in diverse groups of the present study.

Time	W 2					W 4				
Parameters	Sham	MI	MI-DPP	MI-SC	MI-SC/DPP	Sham	MI	MI-DPP	MI-SC	MI-SC/DPP
**Dimensional Parameters:**										
**LVIDd (mm)**	6.45 ± 0.43	7.83 ± 0.41 ****	7.37 ± 0.20 ***	6.88 ± 0.24 ††††	6.67 ± 0.17 ††††‡‡	6.40 ± 0.34	8.88 ± 0.41 ****	7.15 ± 0.17 **††††	6.68 ± 0.24 ††††	6.23 ± 0.20 ††††‡‡‡
**IVSd (mm)**	2.07 ± 0.26	1.52 ± 0.20 ****	1.63 ± 0.17 **	1.73 ± 0.15 *	1.97 ± 0.11 ††‡	2.10 ± 0.26	1.50± 0.13 ****	1.73 ± 0.15 *	1.90 ± 0.08 ††	1.97 ± 0.11 †††
**LVPWd (mm)**	1.70 ± 0.21	1.90 ± 0.31	1.88 ± 0.13	1.77 ± 0.11	1.68 ± 0.11	1.75 ± 0.10	2.13 ± 0.29 *	1.80 ± 0.13 †	1.68 ± 0.20 ††	1.63 ± 0.11 †††
**LVIDs (mm)**	3.60 ± 0.22	5.15 ± 0.28 ****	4.53 ± 0.15 ****††	3.98 ± 0.21 ††††‡	3.80 ± 0.16 ††††‡‡‡	3.70 ± 0.26	6.70 ± 0.49 ****	4.30 ± 0.13 **††††	3.97 ± 0.20 ††††	3.78 ± 0.29 ††††‡
**IVSs (mm)**	2.02 ± 0.11	1.70 ± 0.21 *	1.72 ± 0.14 *	1.82 ± 0.26	1.95 ± 0.10	2.07 ± 0.20	1.47 ± 0.14 ****	1.73 ± 0.07 **	1.78 ± 0.11 *†	1.93 ± 0.07 †††
**LVPWs (mm)**	2.48 ± 0.16	1.95 ± 0.17 ****	1.95 ± 0.19 ****	2.10 ± 0.08 **	2.15 ± 0.13 *	2.27 ± 0.17	1.60 ± 0.24 ****	1.92 ± 0.23 *†	2.15 ± 0.10 ††††	2.08 ± 0.11 †††
**LVM (mg)**	667.9 ± 117.47	787.52 ± 123.24	741.62 ± 65.57	660.78 ± 46.03	668.71 ± 58.52	678.45 ± 63.15	1052.83 ± 134.25 ****	711.89 ± 55.78 ††††	652.32 ± 51.27 ††††	590.92 ± 54.69 ††††
**Flow Parameters:**										
**EF (%)**	86.73 ± 1.79	57.6 ± 2.88 ****	60.7 ± 1.33 ****	66.05 ± 3.83 ****†††‡	76.1 ± 2.34 ****††††‡‡‡‡####	84.82 ± 2.22	54.48 ± 5.09 ****	65.97 ± 2.74 ****††††	72.67 ± 2.11 ****††††‡‡	80.32 ± 1.30 ††††‡‡‡‡##
**FS (%)**	48.53 ± 1.74	32.73 ± 4.01 ****	33.05 ± 2.69 ****	38.03 ± 2.81 ****†‡	42.48 ± 2.23 **††††‡‡‡‡	46.68 ± 2.59	31.23 ± 1.05 ****	35.53 ± 2.29 ****	37.95 ± 2.66 ****††	43.55 ± 2.71 ††††‡‡‡‡#

Data are means ± SD. * *p* < 0.05, ** *p* < 0.01, *** *p* < 0.001, and **** *p* < 0.0001 vs. sham; † *p* < 0.05, †† *p* < 0.01, ††† *p* < 0.001, and †††† *p* < 0.0001 vs. MI; ‡ *p* < 0.05, and ‡‡ *p* < 0.01, ‡‡‡ *p* < 0.001, and ‡‡‡‡ *p* < 0.0001 vs. MI-DPP; # *p* < 0.05, ## *p* < 0.01, and #### *p* < 0.0001 vs. MI-SC.

## Data Availability

The original data presented in the study are included in the article; further inquiries can be directed to the corresponding author.
